# Editorial: Exploring new interventions in myocardial remodeling: from experimental to clinical studies

**DOI:** 10.3389/fphys.2023.1264279

**Published:** 2023-08-04

**Authors:** Andrey Jorge Serra, Stella de Sousa Vieira, Luis Felipe Neves Dos Santos

**Affiliations:** ^1^ Medicine Department, Federal University of São Paulo, São Paulo, Brazil; ^2^ College of Veterinary Medicine, Purdue University, West Lafayette, IN, United States

**Keywords:** heart-drug effects, cardiac remodeling, myocardia ischemia, cardiac disease, cardiac dysfunction, exercise

This Research Topic targeted studies dealing with pathological myocardial remodeling (MR). Therefore, we considered evaluating manuscripts that contribute to the knowledge of the pathophysiology of RM. In addition, we encouraged the submission of manuscripts addressing multitarget approaches to MR therapy. At the end of the review process, four articles were accepted for publication in the journal.

In a mini review, Fonsecas and Izar clarified the role of inflammation in post-infarction MR. This is an interesting review that allows us to understand the role of inflammation in disease progression and shows that anti-inflammatory therapies can reduce major cardiovascular outcomes. The authors have assembled a body of evidence that only therapies that act on the NLRP3 inflammasome or interleukin 1 beta have cardiovascular disease benefits. This notion is based on findings that inflammasome (NLRP3) activation, subtypes of lymphocytes, interleukin 6, and some inflammatory biomarkers are associated with greater infarct size and impaired ventricular function after myocardial infarction (MI).

Exercise training is one of the most important cardioprotective interventions (Serra et al., 2008; Veiga et al., 2019; Torres et al., 2020). The benefits of physical rehabilitation intervention in infarcted patients have been widely reported (Back et al., 2023; Murata et al., 2023), but little is known about the effects of exercise on myocardial responsiveness after infarction (Veiga et al., 2020). In a brief research report, Portes et al. examined the role of swimming training on myocardial responsiveness to infarcts of different sizes. The MI was induced by permanent coronary artery occlusion in female Wistar rats. After 4 weeks MI, surviving animals were subjected to swimming training of 60 min/day for 5 days/week for an additional 8 weeks. Working with *in vitro* papillary muscles derived from the left ventricle, the authors documented significant depression of myocardial inotropism and lusitropism at different calcium concentrations. Exercise training attenuated the deleterious effects of MI, particularly in relation to the maximal positive and negative values of the first temporal derivative and the time to peak tension. In addition, exercise training attenuated the decrease in myocardial responsiveness proportional to the size of MI.

It is known that cardiovascular risk factors and stage of disease can influence the phenotype of monocyte subpopulations (Patterson et al., 2021). In a clinical trial, Carvalho et al. examined the effects of lipid-lowering agents and antiplatelet agents on monocyte subpopulations in patients with acute myocardial infarction (AMI). The study included patients treated with pharmacological thrombolysis in the first 6 h of a ST segment elevation myocardial infarction (STEMI). Before the invasive procedure, patients were randomised to rosuvastatin 20 mg or simvastatin 40 mg plus ezetimibe 10 mg and ticagrelor 90 mg or clopidogrel 75 mg, in addition to routine therapy for AMI. At six-month follow-up, the study showed a higher percentages of classical monocytes (i.e., CD24++ and CD16^−^) and a lower proportion of nonclassical monocytes (i.e., CD14^+^CD16++) compared with baseline analysis. Furthermore, modulation of the chemokine receptors CCR2, CCR5, and CX3CR1 by different classes of monocytes was altered after infarction, with no apparent effect of pharmacological therapy. Overall, the data from this study demonstrate that the inflammatory phenotype of monocytes in infarcted patients persists even with lipid-lowering and antiplatelet therapy.

Finally, Silva et al. carried out an experimental study to investigate the structural, functional, and genetic adaptation of cardiac pressure overload induced by pulmonary artery banding (PAB) in rats. An important contribution of the study was to document changes over time in PAB to understand the evolution of ventricular remodeling and to identify transitions from a physiological adaptation process to a state of maladaptation. Male Wistar rats were subjected to a well-documented method of inducing PAB (de Melo et al., 2016) and observed for up to 8 weeks. The authors reported right ventricular (RV) hypertrophy independent of assessment time, but myocardial fibrosis was more pronounced with longer duration. In addition, higher expression of genes related to hypertrophy and extracellular matrix was observed in the initial subgroups and apoptosis genes in the PAB subgroups with longer follow-up in the RV. Interestingly, the left ventricle (LV) was affected by increased RV afterload, as evidenced by a higher percentage of fibrosis and altered expression of genes related to various functions, particularly hypertrophy.

In conclusion, in this Research Topic, manuscripts have been gathered aiming to explore physiological and therapeutic aspects for various causes of adverse cardiac remodeling. We hope that the research topics will reach readers who specialize in fields other than heart disease.
